# Cost-effectiveness of cetuximab and panitumumab for chemotherapy-refractory metastatic colorectal cancer

**DOI:** 10.1371/journal.pone.0175409

**Published:** 2017-04-12

**Authors:** Adriana Camargo Carvalho, Frederico Leal, Andre Deeke Sasse

**Affiliations:** Centre for Evidence in Oncology, Department of Internal Medicine, University of Campinas (UNICAMP), Campinas, Sao Paulo, Brazil; University of Crete, GREECE

## Abstract

**Background:**

Cetuximab and panitumumab are monoclonal antibodies targeting the epidermal growth factor receptor. Both drugs are active against RAS wild type metastatic colorectal cancer after chemotherapy failure, with similar efficacy and toxicity profiles. However, their cost and limited survival benefits may compromise incorporation in the Brazilian public healthcare system, the Unified Heath System (Sistema Único de Saúde) (SUS).

**Methods:**

A cost-effectiveness analysis was conducted using a Markov model from the Brazilian Public health perspective and a lifetime horizon in patients with RAS -wt mCRC. Transition probabilities and mortality rates were extracted from randomized studies. Treatment costs were obtained from price tables regulated by the Brazilian Health Ministry. The World Health Organization recommendation of three times GDP per capita was used to define the cost-effectiveness threshold.

**Results:**

The use of cetuximab or panitumumab for chemotherapy-refractory mCRC patients resulted in 0.22 additional life-years relative to BSC, with incremental cost-effectiveness ratios (ICERs) of $58,240 and $52,772 per LY, respectively. That exceeds the pre-specified threshold for cost-effectiveness. Acquisition of biological agents was the major driver of increased costs.

**Conclusions:**

Our economic evaluation demonstrates that both cetuximab and panitumumab are not a cost-effective approach in RAS-wt mCRC patients. Discussion about drug price should be prioritized to enable incorporation of these monoclonal antibodies in the SUS.

## Introduction

Colorectal cancer (CRC) causes more than 600,000 deaths per year worldwide and it is the third most common cause of cancer death. In Brazil, about 32,600 people are diagnosed with CRC, causing around 14,000 deaths each year[1]. About 25% of patients have metastatic disease (mCRC) by the time of diagnosis, and up to 50% will develop metastases at some point[2].

Although some patients with liver metastasis may be treated by curative resection, mCRC is an incurable disease in most cases. Hence, treatment strategies should focus on improving survival and symptom control[3]. In the last two decades, a better understanding of the development and progression of mCCR translated into more effective treatments, resulting in increased median survival from 6 to 22–24 months[4]. Cytotoxic chemotherapy is the mainstay treatment for mCRC[3], but novel targeted therapies have also played a role in survival improvement and disease control [5].

Cetuximab and panitumumab are monoclonal antibodies (MoAbs) that target the epidermal growth factor receptor (EGFR) extracellular domain, and inhibit its signaling. Treatment with these drugs may be delivered in combination with chemotherapy, or as monotherapy after chemotherapy failure. Both strategies have shown survival improvements for mCRC[6–8]. However, their benefit is limited to patients with *RAS* wild-type (wt-*RAS*) tumors [9–11]. Recently published data shows that panitumumab is non-inferior to cetuximab as monotherapy after chemmotherapy failure. The two drugs are similar with regard to overall survival (OS) and progression-free survival (PFS), with only a small difference in toxicity[12].

Cost-effectiveness of these agents in Brazil is unknown and none of the alternatives are currently available for patients in the Brazilian Unified Health System (Sistema Único de Saúde) (SUS). Thus, in the public system, a patient whose disease has progressed to different chemotherapy regimens usually receives best supportive care (BSC) alone. Alternatively, in recent years, the MoAbs are compulsorily provided by the Department of Health as consequence of lawsuits filed by SUS-dependent patients. This practice puts a heavy burden on the public budget and inequality of access to better therapies[13] [14]. For the SUS premise to be respected, the incorporation of new drugs should be based on assessments of effectiveness and cost-effectiveness to optimize the use of public resources and the efficiency of the health system as a whole[15]. The aim of this study was to evaluate the cost-effectiveness of cetuximab and panitumumab compared with BSC, for chemotherapy-refractory mCRC from the perspective of the SUS.

## Material and methods

### Overview and treatment strategies

A cost-effectiveness analysis was developed to estimate lifetime outcomes and costs in a hypothetical cohort of patients with chemotherapy-refractory wt-*RAS* mCRC. This economic analysis evaluated three different strategies: (1) panitumumab until treatment failure, then BSC; (2) cetuximab until treatment failure, then BSC; (3) BSC alone.

Survival data stratified by wt-*RAS* status was extrapolated from the CO.17 study [16], a randomized trial that compared cetuximab versus BSC as third-line treatment for mCRC. The efficacy of panitumumab was considered similar to cetuximab, according to results of the ASPECCT trial, which compared outcomes of both anti-EGFR MoAbs in the same setting[12].

### Model structure

TreeAge Pro Suite 2015 software was used to build a Markov model to reflect the natural history of mCRC and current standard of care. Patients were moved between predetermined health states according to transition or recursion probabilities, mutually excluded, in quarterly cycles. This arbitrary interval was chosen because it reproduces the usual pattern of package and payments for mCRC management in Brazil.

**Fig 1:**
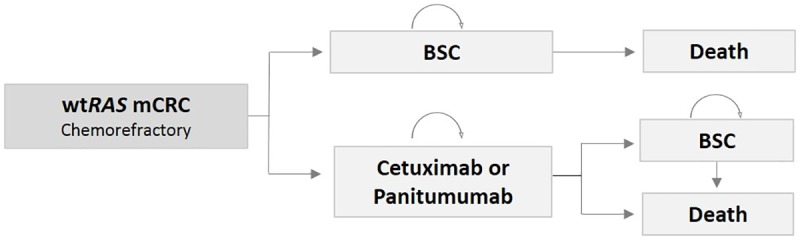
Markov model structure. BSC, best supportive care; mCRC, metastatic colorectal cancer; wt*RAS*, wild type RAS.

As seen in Fig 1, after the beginning of treatment with either MoAb, patients faced the following possibilities: (1) remain on treatment: (2) progress to BSC or (3) die. For those patients receiving BSC, two health conditions were possible: (1) remain on BSC or (2) die.

The probability of transitioning from one state to another was estimated from PFS and OS curves using a log-linear regression model. To capture all relevant differences in costs and outcomes of the alternatives analyzed, the temporal horizon adopted was a lifetime period, that is to say, until death of all patients in the model.

### Economic assumptions

This cost-effectiveness analysis was conducted from the perspective of a Brazilian universal health care system as a paying source. Considering that there is no cost-effectiveness threshold established for the Brazilian scenario, the limit of three times Gross Domestic Product (GDP) per capita for each life year (LY) gain represented, hypothetically, a good value for money, as recommended by the Commission on Macroeconomics and Health of World Health Organization (WHO) [17]. The GDP per capita in Brazil was equivalent to $8,250 USD in 2015, according to official Finance Ministry data[18].

For the different approaches, the incremental cost-effectiveness ratio (ICER) was the primary outcome, calculated by dividing the incremental cost difference between strategies by the incremental life expectancy. Results were presented as the added cost in US dollars for each year of life gained with each strategy (cost/life-year saved). The exchange rate considered was $3.50 BRL = $1 USD. Future costs and benefits had an annual discount of 5%.

### Costs

Since the criteria set were met, and patients underwent any of the strategies, costs of feasibility and treatment monitoring were accounted for. Considering the limits of coverage by SUS, we estimated only direct and relevant costs, incurred by therapeutic response control and clinical toxicity management. Nonmedical and indirect costs were not included in this model. The entire target population was assumed to have been been previously screened for the RAS mutation status of their tumor. Thus, the cost of RAS mutation screening did not differ between the treatment arms and was disregarded.

Costs related to the MoAb acquisition were extracted from the Maximum Price of Sale to the Government (PMVG) 2016 table, including the Brazilian state excise duty 0%. This base case model was designed for a frequency of one application every two weeks, according to therapeutic strategies and patient characteristics presented in Table 1.

**Table 1:** Anti-EGFR antibody doses estimated for biweekly consumption in base case mCRC treatment.

Monoclonal Antibody	Cetuximab	Panitumumab
Doses	500 mg/m^2^	6 mg//Kg
Average weight or body surface	1.75m^2^	70 Kg
Total estimated dose	875 mg	420 mg
Unit vial price (100 mg)	$166.70	$270.43
Total Cost Per Application	$1,500.30	$1,352.11

The total cost of each treatment was estimated from official price lists regulated by the Health Ministry and included drugs and administration costs, adverse event management, routine laboratory tests, radiological examination, usual medical care, and professional fees (Table 2). Frequency of procedures and treatment protocols were standardized and based on experts’ panel opinion to reflect the current clinical practice in the SUS.

**Table 2:** Direct quarterly estimated costs for each health state evaluated, in US dollars ($).

Resource	BSC	Panitumumab	Cetuximab
MoAb	0	8,112.70	9,001.80
Outpatient appointment	27.63	27.94	27.94
Laboratory tests	11.86	15.68	15.68
Imaging	2.71	118.19	118.19
Hospitalization	104.98	168.13	171.82
Total Cost	147.18	8,442.82	9,335.43

MoAb, monoclonal antibody; BSC, best supportive care; Outpatient appointment, Medical, Nutrition and Psychology; Laboratory tests, complete blood count, carcinoembryonic antigen, creatinine, aspartate aminotransferase, alanine aminotransferase, calcium, bilirubin; Imaging, CT scans, chest X-ray and abdomen ultrasound.

Table 3 summarizes the frequency of adverse events extracted from the ASPECCT trial. Only grades 3 and 4 adverse events, which need hospitalization, were included, because the management of all outpatient adverse events are typically associated with out-of-pocket costs. The reimbursement of expenses during the hospitalization in the SUS operates by a system in which the hospital receives a predetermined value for each period or day of stay, regardless of procedures or healthcare resources used by the hospital. These values were collected from the SUS Management System Table of Procedures, Medications and OPM Orthoses, Prostheses and Special Materials (Sistema de Gerenciamento da Tabela de Procedimentos, Medicamentos, Órteses e Próteses e Materiais Especiais) (SIGTAP) database.

**Table 3:** Estimated probability and direct costs of grade 3 and 4 adverse events for a 3-month interval, which are covered by health public system through hospitalization.

Adverse Events Grades 3 or 4	Panitumumab probability of occurrence	Cetuximab probability of occurrence
Diarrhea	0.020	0.018
Abdominal pain	0.034	0.028
Vomiting	0.018	0.014
Anemia	0.026	0.030
Dyspnea	0.010	0.014
Upper respiratory tract infection	0.004	0
Infusion reactions	0.002	0.018
Average cost per quarterly cycle	$63.34	$66.84

### Outcome parameters

The transition probabilities between health states of patients on BSC or treated with MoAbs were calculated using data of PFS and OS curves obtained from the National Cancer Institute of Canada Clinical Trials Group CO.17 trial [16]. Data safety was obtained from ASPECCT study [12]. For each 3-month interval, patients treated with cetuximab or panitumumab had a 20% probability of dying and 29% probability of progressing. The need for dose reductions in the panitumumab and cetuximab groups were respectively 17.5% and 18%. By contrast, in each cycle, patients in BSC had a 36% probability of dying and a 64% probability of remaining in BSC.

### Sensitivity analysis

A tornado diagram was used to assess the robustness of results and the uncertainty associated with main parameters used to build the model. The variables tested were the probabilities of progression, dose reductions and death, unit values of vials and fluctuations on body surface area and weight of patients undergoing to MoAbs. The outcome parameters were varied from -25% to + 25% of the original baseline value. In these analyses, the cost of MoAbs was varied from zero (hypothetically) to the current value in an effort to discover the expected vial price, which makes each strategy cost-effective (Table 4). Based on results of the tornado analysis, we performed one-way deterministic sensitivity analyses, which had significant impacts on the ICER. The goal was to define the ideal price of MoAbs in the current SUS.

**Table 4:** Baseline values in the decision analysis model and their range in the sensitivity analysis.

Variable	Baseline value	Range in Sensitivity Analysis
Transition probability from treatment to BSC	0.29	0.22–0.36
Transition probability from treatment to death	0.20	0.15–0.25
Need for cetuximab dose reductions	0.18	0.14–0.23
Need for panitumumab dose reductions	0.18	0.13–0.22
Cetuximab 100mg vial cost (per unit)	166.70	0–166.70
Panitumumab 100mg vial cost (per unit)	270.42	0–270.42
Body surface	1.75m^2^	1.60–1.90m^2^
Body weight	70kg	50–90kg

## Results

### Benefits

Patients undergoing BSC had an estimated life expectancy around 6.6 months. The incorporation of cetuximab or panitumumab resulted in 0.22 LY (2.64 months) incremental survival over BSC, reaching a little further at 9 months.

### Incremental costs

Treatment with BSC generated an average cost of $429.13, while a patient undergoing treatment with panitumumab or cetuximab cost $11,859.04 and $13,043.32, respectively. The incremental cost generated by incorporation of cetuximab over panitumumab was approximately $1,184.28 per patient.

### Cost-effectiveness

The ICER was $52,771.92 per LY for panitumumab and $58,239.76 per LY for cetuximab. That means cetuximab exceeded by 135% the three GDP per capita limit of $24,751, while panitumumab exceeded such limit 113% over the hypothetical threshold. Thus, neither antibody can be considered cost-effective from a SUS perspective with the registered prices. However, panitumumab was dominant over cetuximab (Table 5).

**Table 5:** Cost-effectiveness rankings from SUS perspective.

Strategies	Cost ($)	IncrementalCost ($)	Effectiveness	Incremental Effectiveness	ICER ($/life-year saved)
BSC	429	-	0.55	-	-
Panitumumab	11,859	11,430	0.77	0.22	52,772
Cetuximab	13,043	12,614	0.77	0.22	58,240

ICER, Cost-Effectiveness Incremental Ratio; BSC, best supportive care.

### Sensitivity analysis

**Fig 2:**
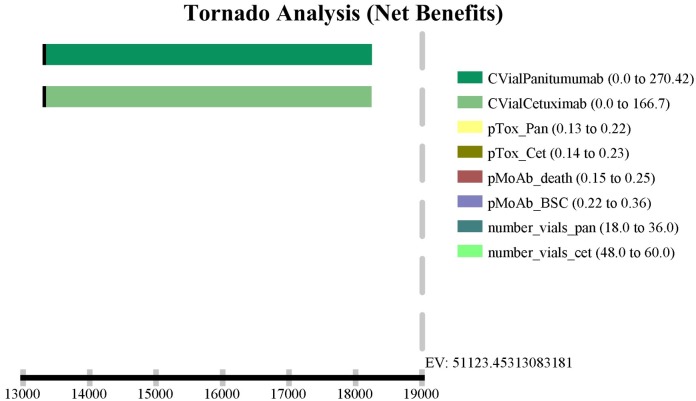
Tornado diagram showing influence of costs with vial acquisition. CVialPanitumumab, cost of panitumumab vial; CVialCetuximab, cost of cetuximab vial; pTox_Pan, probability of reduced dose due to toxicity of panitumumab; pTox_Cet, probability of reduced dose due to toxicity of cetuximab; pMoAb_death, probability of death using monoclonal antibodies; pMoAb_BSC, probability of progression using monoclonal antibodies; number_vial_pan, number of panitumumab vials; number_vial_cet, number of panitumumab vials.

The sensitivity analysis conducted with major outcomes and cost parameters indicated that the ICER was sensitive only to variation in the MoAbs vials costs (Fig 2). None of the ICERs were changed significantly when other parameters varied within the assigned ranges.

**Fig 3:**
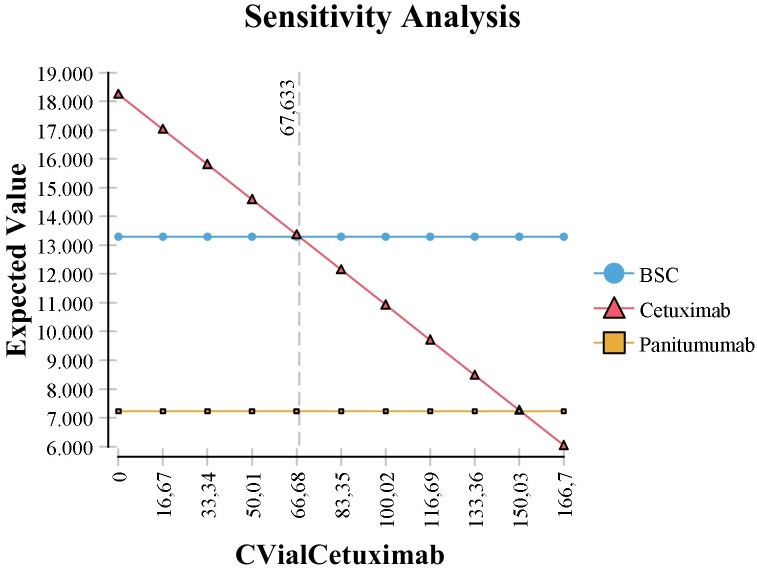
One-way deterministic sensitivity analysis related to price of 100 mg Cetuximab vial. CVialCetuximab, Cost of vial cetuximab; BSC, best supportive care; WTP, willingness to pay.

**Fig 4:**
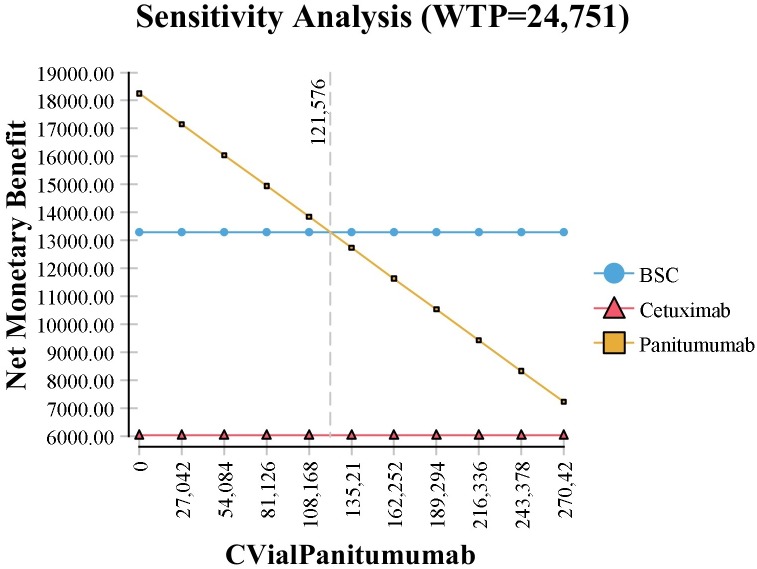
One-way deterministic sensitivity analysis related to price of 100 mg Panitumumab vial. CVialPanitumumab, Cost of vial panitumumab; BSC, best supportive care; WTP, willingness to pay.

The one-way deterministic sensitivity analyses showed that, considering a threshold value of $24,751, the cost of cetuximab and panitumumab vials would need to fall approximately 60% (Fig 3) and 55% (Fig 4) respectively, from the current price to be cost-effective.

## Discussion

This paper aimed to evaluate the cost-effectiveness of anti-EGFR antibodies, cetuximab and panitumumab, for the treatment of colorectal cancer from the prospective of the Brazilian public health system.

The treatment with cetuximab or panitumumab plus BSC have clinically and statistically significant advantages over treatment with BSC alone for patients with wt*RAS* mCRC that progressed after standard chemotherapy. The evidence derived from clinical studies suggests benefits not only in overall survival but also a longer period of time before significant physical deterioration. However, the challenge of selecting the best third-line approach for mCRC must not only consider factors such as convenience of handling, quality of life, toxicity and response rate [19, 20], but also costs. Novel targeted agents may add significant costs to cancer treatment, since these drugs are usually more expensive than cytotoxic chemotherapy [21]. The economic impact resulting from the higher oncology cost curve has worried economists and managers, raising complex issues around the world regarding the sustainability of health systems and the ability to provide efficiently and equitable care [22].

In the Brazilian public health setting, most patients do not receive MoAb treatment in combination with chemotherapy in first- or second-line treatment due to cost and system constraints. Recent data from the Ministry of Health in Brazil show significant and growing expenses related to the supply of high cost drugs, which are not included in the SUS coverage list. This supply occurs either through administrative channels or by an indiscriminate “judicialization” of the right to health, guaranteed by the Constitution. In this context there are no current Brazilian studies being developed to evaluate the economic consequences and how many wt-*RAS* mCRC patients have been benefited by this treatment. It is not clear if the incorporation of anti-EGFR therapy could be more favorable in terms of equal access and the lower budgetary impact the effectiveness, safety and economic aspects involved in this technology incorporated by SUS.

If we believe that the limit of three times the GDP per capita for each life-year gained with a technology is good value for money in Brazil, according to our model, neither MoAb achieved this cost-effectiveness threshold, which was $24,751 USD in 2015. In the face of similar benefits and safety profiles presented in the ASPECCT trial (cetuximab vs panitumumab: PFS HR,1.00 [95% CI, 0.88–1.14]; OS HR, 0.97 [95% CI,0.84–1.11]), this cost-effectiveness analysis revealed panitumumab to be less costly per life-year gained, so, preferred relative to cetuximab in chemotherapy refractory setting.

Despite the existence of *RAS* as a biomarker for selecting possible responders, one would require other predictive markers to select patients prone to experience a more robust clinical benefit[23]. Future perspectives for breaking of a patent protection applied to the reference MoAbs and the development of biosimilars with cheaper prices, could contribute a significant impact on the findings of this study as our sensitivity analysis indicates that our model is sensitive mainly to the drug costs. Until this occurs and while there is no evidence of better clinical outcomes, only a reduction in price by 60% for cetuximab and 55% for panitumumab could lead to favorable ICERs in the current Brazilian public health scenario.

Our results are aligned with the NICE (National Institute for Health and Care Excellence) 2012 opinion in which both cetuximab (alone or in chemotherapy combination) and panitumumab (monotherapy) after mCRC first-line treatment were not recommended in the United Kingdom (UK) for not providing sufficient benefit to patients to justify the high costs[24].

Some limitations and caveats can be highlighted to better put the results in context. First, efficacy parameters were extracted from international clinical trials, which may not reflect the Brazilian reality for this cohort in its entirety. Also, as mentioned above, targeted therapy benefit may be heterogeneous, with some patients experiencing longer survival than others. Second, this model considered the subpopulation wtRAS mCRC, but was based on data from the CO.17 study that stratified KRAS status. Third, there is no threshold value established for the incorporation of new technologies in the Brazilian public setting and the hypothetical limit recommended by WHO might not represent the wish of most stakeholders. The threshold acceptability is subjective and depends on other factors such as political decisions, social values and budget impact. Fourth, the economic input is based on reference and reimbursement lists and might not fully represent regional and/or specific cost settings in a country of continental dimensions like Brazil. Finally, use of resources such as duration of hospitalization, periodicity of exams and outpatient appointment was estimated by experts panel and not directly or prospectively obtained. However, this study offers important practical information that must be taken into account in the process of adopting the technology. Discussion about drug price should be prioritized to enable incorporation of these MoAbs in the Brazilian public healthcare system.

The broad assumption that anti-EGFR are not cost-effective in the Brazilian Public System should be made with caution. This analysis focused the relative benefits of the antibodies versus BSC. In the larger context, the median survival of patients with metastatic colorectal cancer in the Fluorouracil era, prior to the introduction of irinotecan, oxaliplatin and monoclonal antibodies was about 12 months or less. Nowadays, with all agents used combined or sequentially, the survival of patients has more than doubled to over 30 months. The changing costs over time, derived from the introduction of generic medicines and biosimilar antibodies could alter the results. More important, other cost-effectiveness analysis evaluating the use of anti-EGFR antibodies combined to first-line chemotherapy is warranted.
